# Supplementary material for: A Spin‐Texture Spin‐Valves With Van Der Waals Magnets
Source: Adv Sci (Weinh). 2026 Feb 17;13(25):e00041. doi: 10.1002/advs.202600041 (PMC13137797; doi:10.1002/advs.202600041)
Supplement: Supplementary file 1 — Supporting File: advs74507‐sup‐0001‐SuppMat.pdf. [file ADVS-13-e00041-s001.pdf]

Supplementary Information

A Spin-Texture Spin-valve with van der Waals Magnets

Bing Zhao[1], Roselle Ngaloy[1], Lars Sjöström[1], Saroj P. Dash[1,2,3*]

[1]Department of Microtechnology and Nanoscience, Chalmers University of Technology, SE-41296, Göteborg, Sweden

[2]Wallenberg Initiative Materials Science for Sustainability, Department of Microtechnology and Nanoscience, Chalmers University of Technology, SE-41296, Göteborg, Sweden.

[3]Graphene Center, Chalmers University of Technology, SE-41296, Göteborg, Sweden.

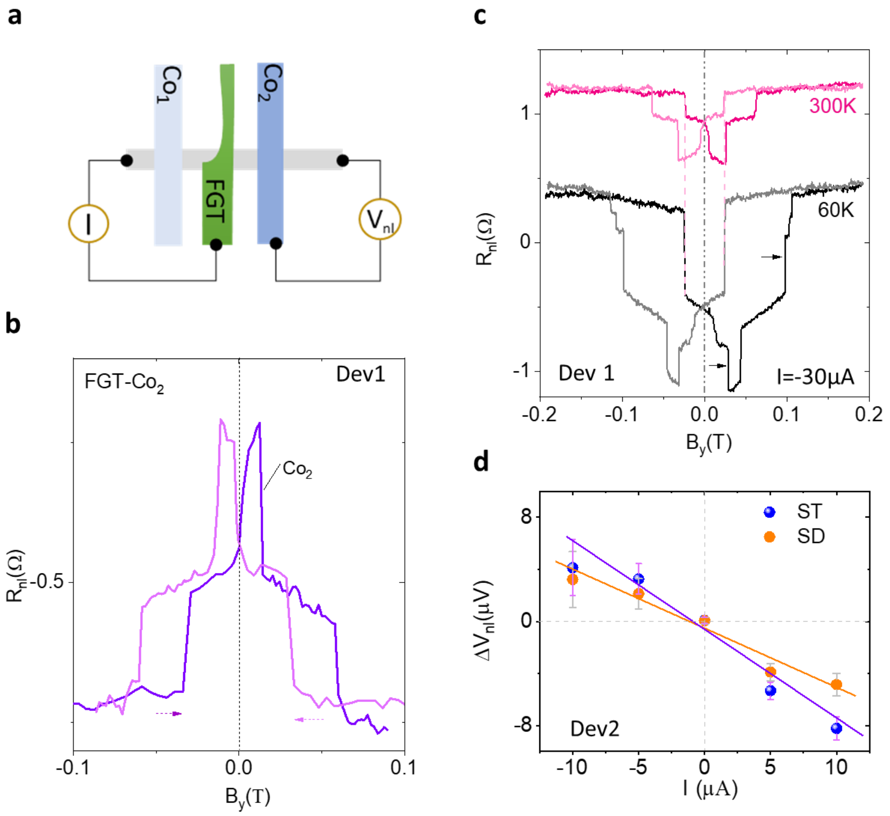

Supplementary Figure S1. a, b. *Schematic for the measurement geometry and measured spin-texture valve signals with another detector Co_2 of Dev 1.* ***c.*** *Temperature-dependent spin-valve signals at 60 K and 300 K of Dev 1 at a bias current of -30 μA.* ***d.*** *Bias dependence of the SD and ST components in Dev 2.*

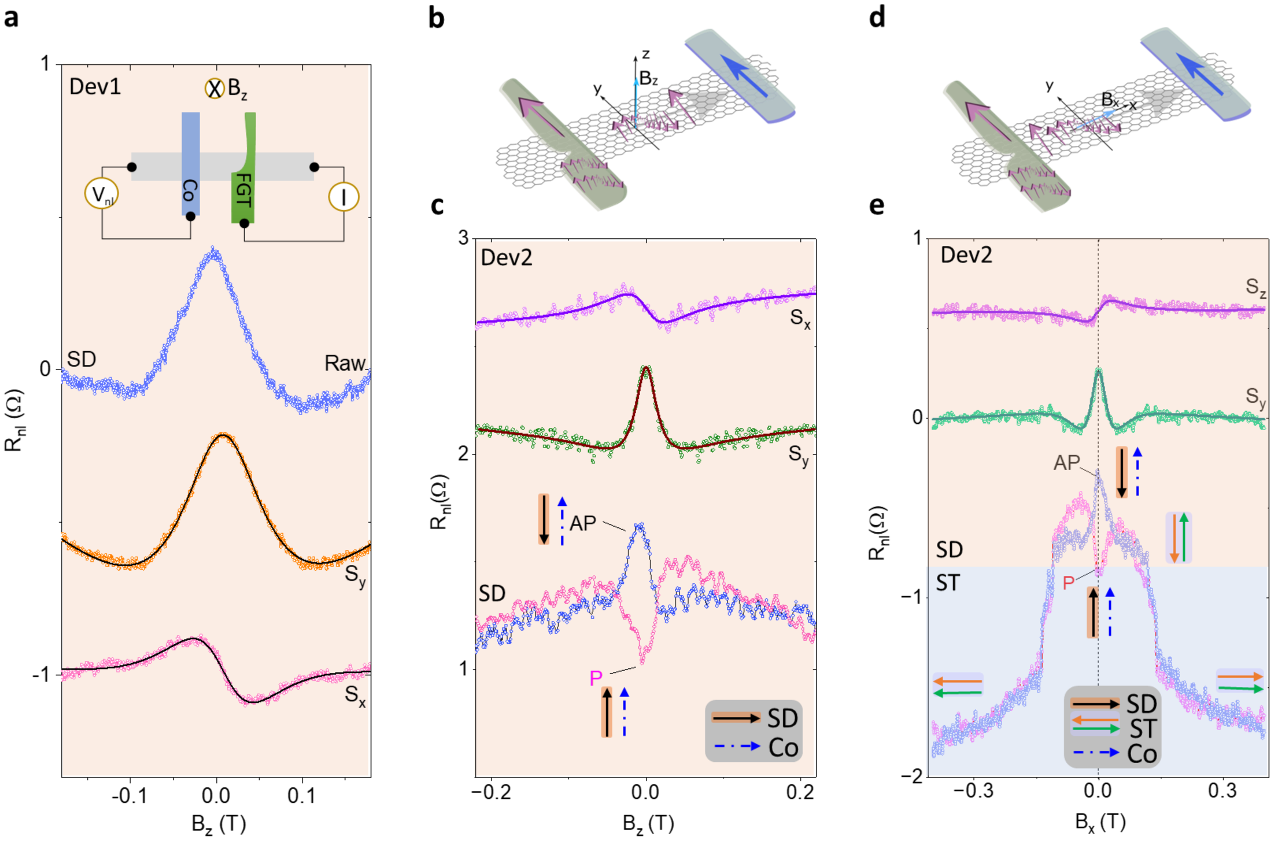

Supplementary Figure S2. Hanle spin precession measurements of spin-texture spin valve devices. a. *Nonlocal zHanle signals with raw data and the symmetric and anti-symmetric components of Dev 1. The symmetric and antisymmetric signals correspond to the contribution of S_y and S_x spins, respectively. The inset is the zHanle measurement geometry.* ***b, d.*** *Schematics of the z(x)-Hanle measurements in the notched FGT-Co spin valve.* ***c, e.*** *z(x)-Hanle signals in parallel (P) and anti-parallel (AP) magnetic moment configurations of Co and SD of FGT, with the symmetric (S_y) and anti-symmetric ($S_{x(z)}$) components of Dev 2. The solid curves are the Hanle fitting results. Arrows in the insets, as indicated, represent the magnetic moment of ST, SD, and Co.*

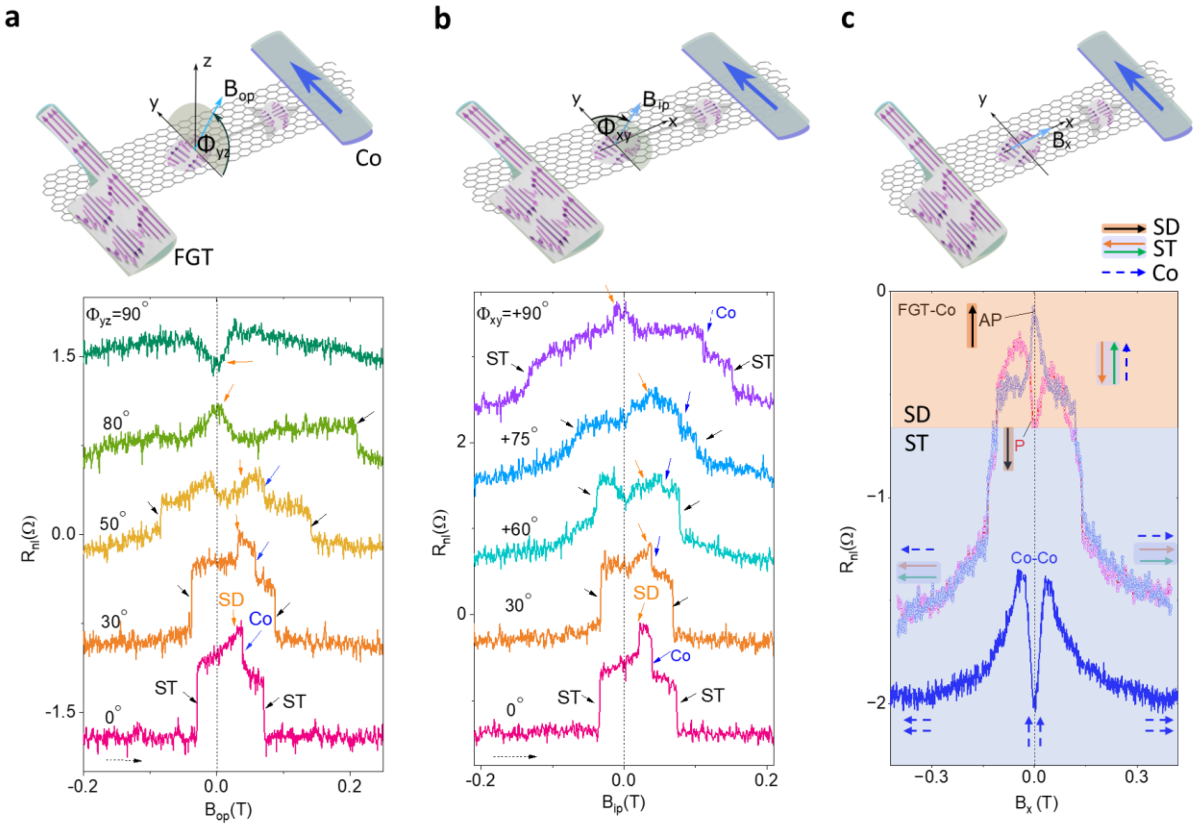

Supplementary ***Figure S3. Angle-dependent measurements of the spin valve signal in Dev 2. a.*** *Schematics and measured data for out-of-plane angle-dependent spin-valve measurement. The magnetic field is applied at an increasing angle in the yz-plane (Φ_{yz}), resulting in an increasing coercive field of the ST component (black arrows) and the Co contact (blue dashed arrows), respectively.* ***b.*** *Schematics and measured data of the in-plane angle dependence of the spin valve signal. As the magnetic field is applied at an increasing angle in the xy-plane (Φ_{xy}), the coercive fields of both the FGT and Co increase.* ***c.*** *Comparison between the raw xHanle signals of FGT-Co with the Co-Co spin valve signal. The Arrows represent the ST (green/orange) and SD (black) domains and Co (blue dashed arrow) reference electrodes.*

Supplementary Note 1. Angle-dependent evolution of the FGT-Co nonlocal spin valve signals

To investigate more details of the evolution of the SD and ST components with the external magnetic field, we performed a series of field sweep measurements at different angles for both out-of-plane (yz plane, Fig. S3a) and in-plane (xy plane, Fig. S3b). The spin signals at different out-of-plane angle Φ_{yz} , change from spin valve signals to the zHanle signal; While the in-plane angle Φ_{xy} related spin signals, evolve continuously from the standard spin valve signals to the xHanle signal. In both scenarios, the ST component shows an increasing coercive field with the angle towards the Hanle measurement position. These detected signals

can be understood as the ST projection along Co following the cos (Φ) relation, which induces an increasing coercive field to fully align the ST domain with the angle Φ_{yz} (Φ_{xy}).

The larger in-plane field B_x continuously rotates both Co and FGT moments to the x-axis (Fig. S3c). However, the xHanle signal of FGT-Co at a larger field range shows much sharper switching than Co-Co electrodes one, suggesting that ST component realignment happened during the Co rotation. This is also supported by the series of in-plane angle Φ_{xy} measurements (Fig. S3b). Noticeably, the xHanle signal of FGT-Co finally saturates with the reference (B_y field) spin valve signal (Fig. 4f in the main text), further confirming the full realignment of both ST and SD components to the Co contact along the x-axis (as indicated in the insets of Fig. S3c).

Supplementary Note 2. Critical features to distinguish the ST from the SD component.

Three critical features can distinguish the ST from the SD component. **(1)** The comparison between the Hanle signal background and that of the B_y-spin valve signal. SD and ST components are present at different backgrounds (see the highlighted ST [light blue] and SD [light orange] area). **(2)** From Fig. 5d and Fig. 5f, one can see that in the z-Hanle and x-Hanle signals, SD dominates the small field range, due to the almost compensated magnetic state of ST character. ST would realign with the external field B_x at a larger field. This is clearly seen from the continuous evolution of the two components (Fig. S3). **(3)** Another difference between SD and ST contributions is the parallel (P) and antiparallel (AP) magnetic alignments of the Co and the SD magnetic states-related Hanle signals in Fig. R3c. The ST component can be removed by $R_{nl}=[R_{nl}(AP)-R_{nl}(P)]/2$, while the SD one will present (see Fig. S2e).

Supplementary Note 3. Spin parameters and spin injection efficiency of Fe_5GeTe_2

To quantitatively describe the spin injection efficiency of Fe_5GeTe_2 in the graphene channel, we computed the spin polarization of FGT in the devices, with the results summarized in Table S1. Since we consider two active components for spin injection, spin polarization P_{SD} and P_{ST} are extracted exclusively for each ST and SD component. The corresponding measurement of $\Delta R_{NL,ST}$ and $\Delta R_{NL,SD}$ are marked in Fig. 1d of the main text.

The spin polarization of Co electrodes (P_{Co}), spin diffusion length λ_{gr}, spin lifetime τ_s are extracted from the reference Co-Co z(x)Hanle measurements[1]. The spin transport parameters in Table S1 are extracted using the Hanle precession-based formula and spin diffusion model[1,2].

$$R_{nl} = \pm \frac{p_i p_d D_s R_{sq}}{W} \int_0^\infty \frac{1}{\sqrt{4\pi D_s t}} e^{-\frac{L^2}{4D_s t}} \cos(\omega_l t)\, e^{-\frac{t}{\tau s}}\, dt \quad \text{(Eq. S1)}$$

where ± sign represents the parallel (antiparallel) magnetization state, W is the width of the graphene channel, R_{sq} is the square resistivity, p_i and p_d are the spin polarizations for the injector and the detector separated at a length of L. D_s and τ_s are the spin diffusion coefficient and spin diffusion time, respectively. The spins in the channel undergo a Larmor precession of frequency $\omega_l = g\mu_B B_z/\hbar$ with Lande g-factor (g = 2) in the perpendicular magnetic field B_z.

Using the formula below, we can extract spin parameters like spin polarization for the ST and SD components of the constricted/notched FGT, respectively[2].

$$\Delta R_{NL,ST(SD)} = P_{FGT,ST(SD)} \cdot P_{Co} \cdot \lambda_{gr} \cdot R_{sq,gr} \cdot \exp\left(-L_{ch}/\lambda_{gr}\right)/(2w_{gr}) \quad \text{(Eq. S2)}$$

Notably, the effective ST spin polarization at zero field is 0 due to its fully compensated magnetic spin texture in FGT. However, the net spin polarization is not zero when the magnetic spin textures are fully aligned. To evaluate the efficiency of ST component injection, a nominal spin polarization P_{ST} = [n(↑↓) - n(↑↑)] / [n(↑↓) + n(↑↑)] is defined, similar to the conventional spin polarization P_{SD} = [n(↑) - n(↓)] / [n(↑) + n(↓)], where n is the density of the spin status. Therefore, the spin polarization P_{SD} and P_{ST} are extracted exclusively for each ST and SD component, respectively. Due to the same Co contact as spin detection, we have a good reason to suppose the spin polarization remains unchanged for Co-Co and FGT-Co nonlocal geometries.

Table S1: Spin parameters extracted from the injection of FGT on the graphene channel at room temperature. $R_{sq,gr}$, L_{ch}, w_{gr}, λ_{gr}, and τ_s are the graphene square resistance, channel length, channel width, spin diffusion length, and spin lifetime τ_s, respectively. RA is the interface resistance of FGT/graphene. $P_{FGT,ST(SD)}$ spin polarization of the ST and SD FGT components.

Devices	$R_{sq,gr}$ (Ω)	L_{ch} (µm)	w_{gr} (µm)	λ_{gr} (µm)	τ_s (ps)	$R_{hetero.}$ (kΩ)	$\Delta R_{nl,tot}$ (Ω)	$\Delta R_{nl,SD}$ (Ω)	$\Delta R_{nl,ST}$ (Ω)	P_{co} (%)	$P_{FGT,SD}$ (%)	$P_{FGT,ST}$ (%)
Dev 1	950	3.4	1.9	2.0	123	4.0	0.59	0.31	0.28	8.1	-6.1	-5.6
Dev 2	818	4.4	2.0	1.9	266	2.7	1.4	0.59	0.84	10.0	-14.3	-20.4

Supplementary Note 4. Magnetic proximity effect in FGT/graphene heterostructure

In devices with FGT/graphene heterostructure, due to proximity, the magnetic properties of the magnet can be acquired by the graphene transport channel. To rule out magnetic proximity effects in devices with FGT/graphene heterostructure, we performed reference measurements using Co-Co (injector-detector) configuration across the heterostructure as shown in Fig. S4a. Spins injected by Co propagate across the FGT/graphene heterostructure and are detected by another Co contact on the other side of the heterostructure (Fig. S4b). In this configuration, the presence of magnetically proximitized graphene can be probed by spin transport measurements, as the interaction of injected spins and induced spin polarization in graphene can cause changes in the measured spin signal.

Hanle precession is a useful tool in probing magnetic proximity, as induced exchange fields can cause additional kinks[3] and/or hysteresis[4] of the Hanle curves for the different sweep directions. However, in our device, no unusual features are observed, indicating the absence of magnetic proximity effect in the heterostructure (Fig. S4c).

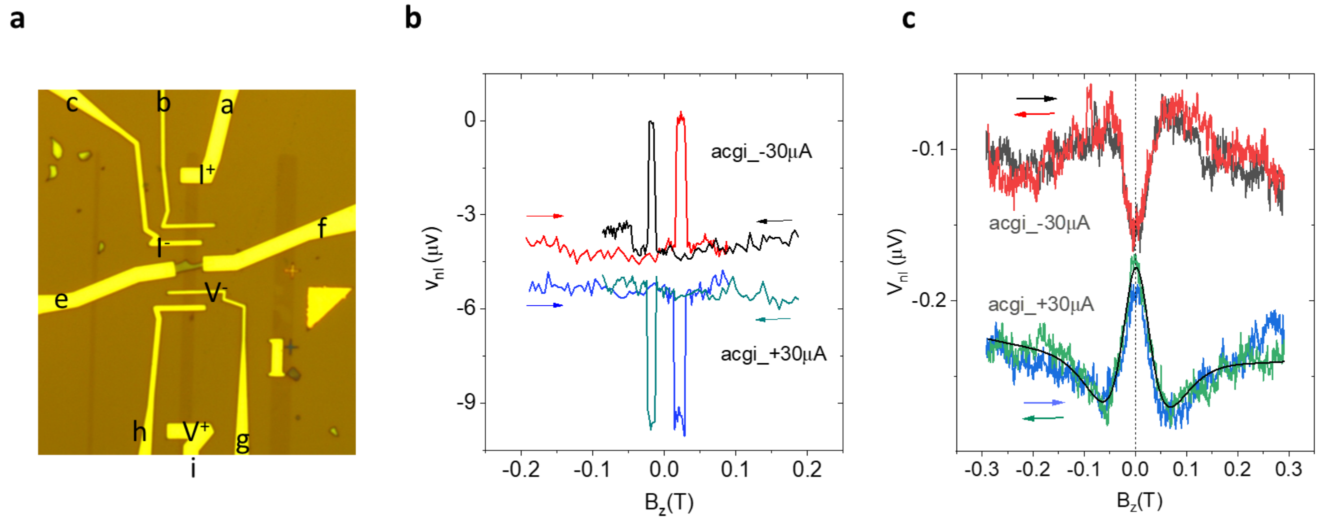

Figure S4. Co-Co reference measurements across FGT/graphene heterostructure for Dev 1. a. *Nonlocal spin valve signal using two Co contacts as spin injector and detector. In the inset, a schematic diagram illustrates the measurement setup.* ***b, c.*** *Spin valve and zHanle precession signals under forward sweep and backward sweep of the magnetic field along y- and z-axis, respectively.*

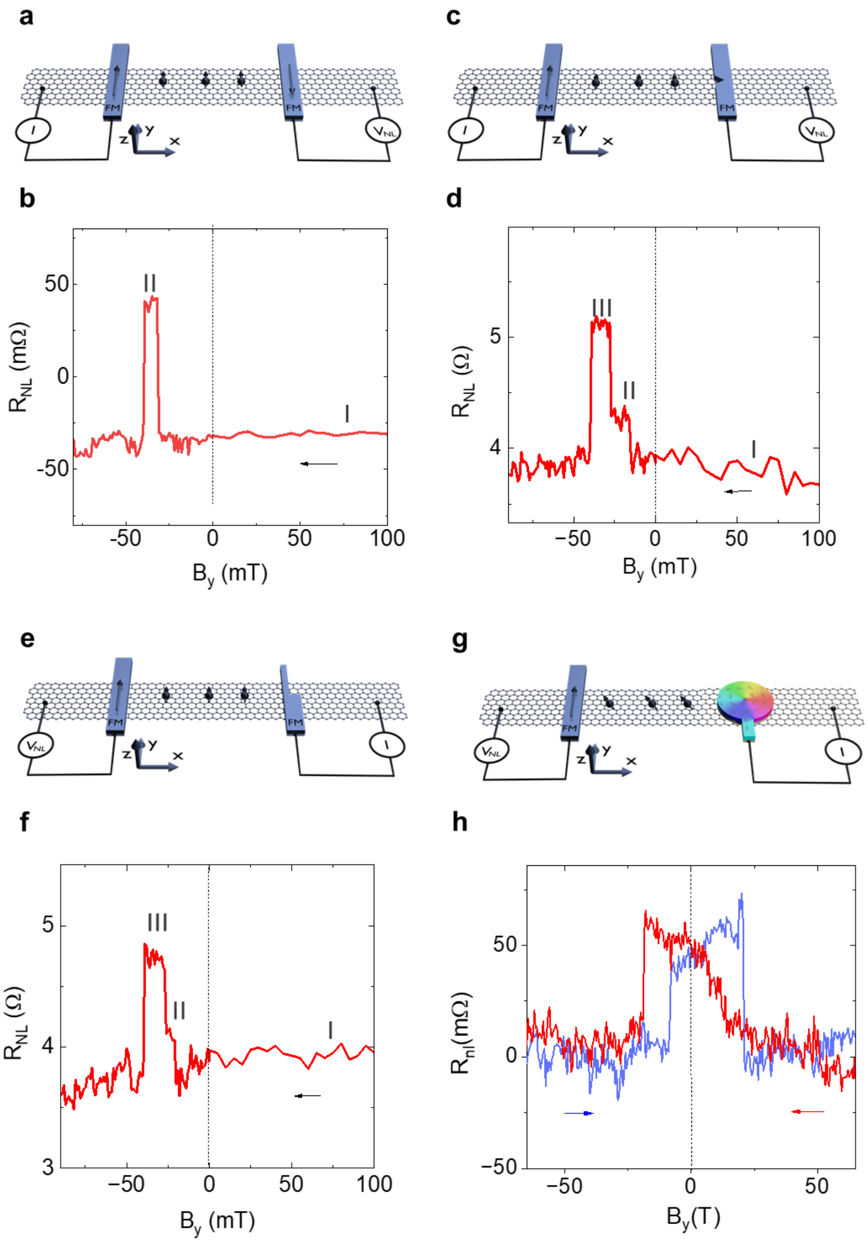

Figure S5. Different Co-Co spin valve geometries and the corresponding spin valve signals. *(**a**) Normal Co contact; (**b**) Notched Co; (**c**) Constrained Co; (**d**) Circular Co. The dashed lines indicate the zero-field point. The arrows indicate the field-sweeping direction. All Reference Co contacts are designated with a narrow-striped shape. To keep it simple, the curves in b), d), and f) only present half-field sweeping results.*

Supplementary Note 5. Factors affecting the spin textures in FGT

It is known that FGT itself hosts exotic spin structures due to Dzyaloshinskii-Moriya interactions (DMI)[5]; however, the spin textures can appear randomly, so it cannot be guaranteed that the spin texture will

always remain at the FGT/graphene region of the spin-valve device. In this work, we utilize the geometrical constraint and notch to nucleate and pin the spin textures at the FGT/graphene interface area. However, some geometric factors of the notch and the constraint may affect the nucleation and pinning efficiency of the spin texture. Systematic optimization of these geometries is essential for real applications. However, to probe the spin textures of 2D magnets with different geometries, we need to pattern them using ion-beam etching. This needs further development, as patterning of FGT cannot be done on a graphene spin-valve device without damaging the graphene channel.

There may be other dimensional issues with spin textures, such as variations in thickness, length, or the angle of the FGT flakes. Our measured devices have thicknesses in the range of 20 to 50 nm, so, the spin textures are more likely to originate from the bulk Dzyaloshinskii–Moriya interaction (DMI) of FGT. Some slight thickness variation may change the spin textures or types, as some works show that the thickness fluctuations across a flake create local variations in magnetic anisotropy and exchange stiffness, which destabilize uniform spin states[6]. Some recent reports also suggest that FGT exhibits a diverse range of magnetic spin structures that depend on its thickness[5]. Other factors, like strain/lattice distortions, can also modify spin–orbit coupling (SOC) and DMI[7,8], and can also be used to tune the spin textures in vdW magnetic materials.

Reference

[1] F. J. Jedema, H. B. Heersche, A. T. Filip, J. J. A. Baselmans, B. J. Van Wees, *Nature* **2002**, *416*, 713.

[2] N. Tombros, C. Jozsa, M. Popinciuc, H. T. Jonkman, B. J. van Wees, *Nature* **2007**, *448*, 571.

[3] J. C. Leutenantsmeyer, A. A. Kaverzin, M. Wojtaszek, B. J. van Wees, *2D Mater.* **2016**, *4*, 014001.

[4] B. Karpiak, A. W. Cummings, K. Zollner, M. Vila, D. Khokhriakov, A. M. Hoque, A. Dankert, P. Svedlindh, J. Fabian, S. Roche, S. P. Dash, *2D Mater.* **2019**, *7*, 015026.

[5] A. K. Gopi, A. K. Srivastava, A. K. Sharma, A. Chakraborty, S. Das, H. Deniz, A. Ernst, B. K. Hazra, H. L. Meyerheim, S. S. P. Parkin, *ACS Nano* **2023**, DOI 10.1021/ACSNANO.3C09602/ASSET/IMAGES/LARGE/NN3C09602_0004.JPEG.

[6] R. Fujita, P. Bassirian, Z. Li, Y. Guo, M. A. Mawass, F. Kronast, G. van der Laan, T. Hesjedal, *ACS Nano* **2022**, *16*, 10545.

[7] N. S. Gusev, A. V. Sadovnikov, S. A. Nikitov, M. V. Sapozhnikov, O. G. Udalov, *Phys. Rev. Lett.* **2020**, *124*, 157202.

[8] C. Ernandes, L. Khalil, H. Henck, M. Q. Zhao, J. Chaste, F. Oehler, A. T. Charlie Johnson, M. C. Asensio, D. Pierucci, M. Pala, J. Avila, A. Ouerghi, *Nanomaterials* **2021**, *11*, 2921.
